# Regression Based Iterative Illumination Compensation Method for Multi-Focal Whole Slide Imaging System †

**DOI:** 10.3390/s21217085

**Published:** 2021-10-26

**Authors:** Róbert Paulik, Miklós Kozlovszky, Béla Molnár

**Affiliations:** 1 Image Analysis Department, 3DHISTECH Ltd., 1141 Budapest, Hungary; robert.paulik@3dhistech.com; 2 Department of BioTech Research Center, Óbuda University, 1034 Budapest, Hungary; kozlovszky.miklos@nik.uni-obuda.hu

**Keywords:** digital pathology, compensation, illumination, inhomogeneity, field of view, adaptive histogram matching, iterative, polynomial regression, multi-focal, shading correction

## Abstract

Image quality, resolution and scanning time are critical in digital pathology. In order to create a high-resolution digital image, the scanner systems execute stitching algorithms to the digitized images. Due to the heterogeneity of the tissue sample, complex optical path, non-acceptable sample quality or rapid stage movement, the intensities on pictures can be uneven. The evincible and visible intensity distortions can have negative effect on diagnosis and quantitative analysis. Utilizing the common areas of the neighboring field-of-views, we can estimate compensations to eliminate the inhomogeneities. We implemented and validated five different approaches for compensating output images created with an area scanner system. The proposed methods are based on traditional methods such as adaptive histogram matching, regression-based corrections and state-of-the art methods like the background and shading correction (BaSiC) method. The proposed compensation methods are suitable for both brightfield and fluorescent images, and robust enough against dust, bubbles, and optical aberrations. The proposed methods are able to correct not only the fixed-pattern artefacts but the stochastic uneven illumination along the neighboring or above field-of-views utilizing iterative approaches and multi-focal compensations.

## 1. Introduction

The integration of digital imaging in medical diagnostics first started in radiology and, due to the advantages of the digital file, such as the possibility of sharing, integration and archiving, the same request appeared in routine histopathology. The digital revolution started with the introduction of whole slide imaging (WSI) technology in pathology. Different scanner devices creating large files have been introduced, presenting tissue structures in an adequate resolution with a high color fidelity [1]. Nowadays, two main WSI technologies are available which solves the narrow field of view (FOV) problem of traditional optical microscopes [2,3,4,5]. The line scanning process scans the samples along a horizontal line; and in contrast to this, the area scanners use a stop and go mechanism to take images in each microscopic field of view individually. The scanning process results in a series of images, which builds up one full montaged picture of the whole tissue [6]. It is difficult to find an adequate solution for stitching these large sets of images, because the computational time and the quality is a critical step in whole slide imaging.

The area scanning process is quite like the widely used panorama photography and the base of the applied stitching methods are common. The moving camera takes several pictures of one object following a lane path. A software composes them into a large object connecting the small parts into one integrated object. These methods typically use an oversized FOV recording process, which results an overlapping area in each fitting side of the image rectangle. There are four overlapping stripes along all sides of one FOV rectangle in area scanners which contain a few tens or hundreds of common pixel columns and rows. These areas are adequate to create the fine alignment (stitching), and this can be used also for intensity compensation [7].

The images that come directly from the scanner camera output channel contain FOVs presenting heterogeneous intensity even though they were recorded with the same exposition time. This inhomogeneity originates from the structural heterogeneity of the tissue sample and from the optical features of the scanner hardware, such as the rapid stage movement, complex optical path or the camera and illumination characteristics [8,9,10]. Therefore, next to the image stitching process, an intensity compensation is needed. However, there are some special requests related to this compensation task. The scanning time is a critical point because the diagnostic time is limited in the pathology department: while the available expert pathologists are limited, the number of the samples increase dynamically. Accordingly, both the image quality and scanning time are critical points. This situation needs an ultrafast scanning system, to create the digital slide without any delay in an appropriate quality. Stitching operation should be fast-processing with minimal use of the available system resources.

There are existing techniques in the field of digital microscopes or in other fields of science for calibrating the intensity inhomogeneities [11]. For instance, in astronomy, the related methods are known as flat-field correction [12], which is a common method to remove artifacts from the images that are caused by the noisiness of the detector or by distortions of the optical path. Another related area is panoramic photography, where the different exposure times or lens distortions can cause intensity differences between the images [13,14,15]. Nowadays the camera software in mobile phones can create and visualize panoramic photos in real-time [16,17,18]. The problem of uneven illumination also appears with other medical devices like Endoscopes [19] or Magnetic Resonance Imaging (MRI) [20].

In the field of digital microscopy a few studies are researching solutions for the intensity inhomogeneities like the BaSiC tool [21] which is based on low-rank and sparse decomposition methods [22,23]. Some researchers applied compensation for fluorescent illuminated slides [10,24,25,26] while others for brightfield slides [8,9,27] to eliminate the mosaicking of artifacts. A few studies utilized traditional color histogram matching methods [6,28,29,30] which is the base of the proposed method.

Similar research found in the literature can be divided into two groups. Studies in the first group try to improve locally the boundaries of the neighbouring images but do not consider the wider environment. This approach is useful if we only want to compensate the images with their immediate environment, with their distant neighborhood independent from them. The other category of approach is to try to estimate background intensities and optical characteristics from a series of images and then compensate for the images. The latter group does not have local compensation, it cannot compensate the adjacent fields of view relative to each other. Our proposed solutions have both properties, and they are specialized to the field of digital microscopy.

In this article we introduce five approaches which can be used as potential solutions to reduce tile effects of multi-focal fluorescent and brightfield digital slides. After implementation, we investigate the advantages and disadvantages of each approach and make attempt to find an adequate solution that meets to WSI needs.

## 2. Materials and Methods

Routine breast cancer slides were used from the archive of the 1st Department of Pathology and Experimental Cancer Research of the Semmelweis University, Budapest, Hungary. Brightfield and fluorescent stained samples randomly collected. The survey was performed with the permission of the Institutional Review Board and Regional Ethics Committee of the Semmelweis University (permit no. 7/2006).

Slides were digitized using a Pannoramic^TM^ P250 Flash and Pannoramic^TM^ P1000 digital slide scanners (3DHISTECH Ltd., Budapest, Hungary) that utilizes Plan-Apochromat objective with 20× (for IHC) or 40× (for FISH) magnification (Zeiss, Jena, Germany) and a CIS color camera (CIS Corporation, Tokyo, Japan) for brightfield and PCO.edge Scientific Complementary Metal-Oxide Semiconductor camera (PCO, Kelheim, Germany) for fluorescence image acquisition.

The sizes of the digital slides of brightfield samples ranged from 0.8 to 2.4 GB and the number of pixels from 50,000 × 50,000 to 100,000 × 170,000. The sizes of the used fluorescent slides were between 9 and 10 GB; the pixel numbers were around 85,000 × 75,000.

The proposed methods are compensating the image channels independently. It means red-green-blue channels in case of brightfield samples and N channel in case of fluorescent slides. All slides in the validation set have 8 bit per channel bit-depth. The methods are not sensitive to whether the background intensity is black (fluorescent) or white (brightfield).

### 2.1. Histogram Matching

In our first approach we try to compensate the tiles using the classical histogram matching algorithm. Since it calculates a function for the whole intensity range, it can match the nonlinear differences: for example, the intensity change is exponential: $i^{\prime }=i^{0.5}$.

The histogram function for an image with *N* intensities:

(1) $$h\left(i\right)=n_{i},\;\;\;\;\;\;i\in \left[0,\;\ldots ,\;N-1\right]$$

where *n_i_* is the number of pixels with intensity *i* and *N* is the number of intensities (e.g., 256).

The Cumulative Distribution Function (*cdf*) [21] gives the probability that any given pixel from the image has a value less than or equal to *i*:

(2) $$cdf\left(i+1\right)=\frac{\sum_{j=0}^{i}h\left(j+1\right)}{\sum_{j=0}^{N-1}h\left(j+1\right)}$$

For compensation, we calculate the histograms for the two overlapping areas (*h*_1_ and *h*_2_). Following, we calculate their cumulative distribution functions (*cdf*_1_ and *cdf*_2_). For each intensity value (*i*_1_) we find *i*_2_ for which *cdf*_1_*(i*_1_*)* = *cdf*_2_*(i*_2_*)*

(3) $$cdf_{1}\left(i_{1}\right)=cdf_{2}\left(i_{2}\right),\;\;\;\;\;\;i_{1},i_{2}\in \left[0,\ldots ,\;N-1\right]$$

**Figure 1:**
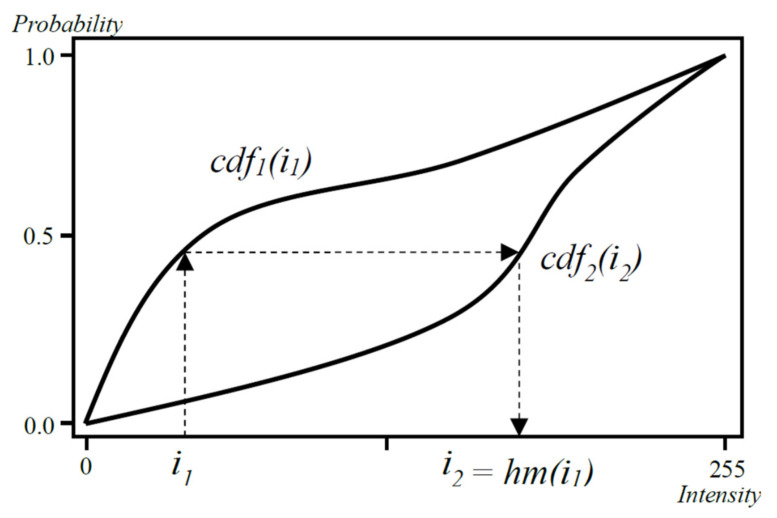
An example of histogram matching: *cdf*_1_ and *cdf*_2_ are the cumulative distribution function, *i*_1_ is the input intensity, and *i*_2_ is the output one.

The result of the histogram matching function (*hm,* Figure 1.):

(4) $$hm\left(i_{1}\right)=i_{2}$$

**Figure 2:**
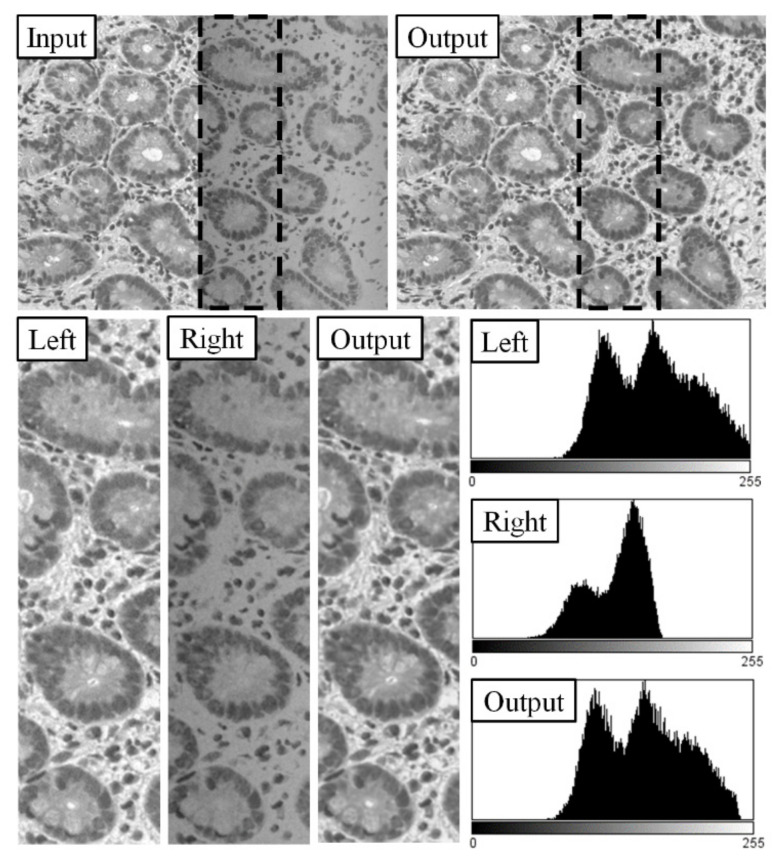
Compensation by histogram matching: first row: (Input) a part of the source FOVs, dashed rectangle: overlap area; (Output) result image compensated by histogram matching; second row: (Left and Right) the overlap areas of the input FOVs and their histograms; (Output) the overlap area of the compensated image and its histogram.

On Figure 2, two neighboring field of views are shown; their original histograms of their overlapping areas (Left and Right); the compensated image (Output) and its histogram. The Output image is transformed from the Right image using compensation to the Left one. The base of this method is when we compensate two images by matching their histograms, the output images will look similar.

### 2.2. Patch Based Histogram Matching

Executing the histogram matching based method we can compensate the differences which are caused by the stochastics of illumination—these differences are homogeneous along the whole area of field of views. However, if the necessary compensation is different on the parts of FOV, a more adaptive method is needed.

The simplest approach would be to execute the histogram matching method for each row [3]. However, in this case, there may not be enough information in each line to execute the matching: if only a few intensities are present, we cannot interpolate and extrapolate since not every intensity has its *cdf* pairs.

As an intermediate solution we can divide the overlap area into patches, where the size of patches are small enough to compensate the differences inside the FOVs but large enough to contain sufficient information for interpolation and extrapolation.

We extend the histogram function for patches:

(5) $$ph_{p}\left(i\right)=n_{p,i},\;\;\;\;\;\;i\in \left[0,\;\ldots ,\;N-1\right]$$

where *n_p,i_* is the number of pixels with intensity *i* in the patch *p* and *N* is the number of intensities. After we calculate the histograms for two neighbouring patches (*ph*_1_ and *ph*_2_), we can calculate their cumulative distribution functions (*cdf_p_*_1_ and *cdf_p_*_2_):

(6) $$cdf_{p1}\left(i_{1}\right)=cdf_{p2}\left(i_{2}\right),\;\;\;\;\;\;i_{1},i_{2}\in \left[0,\ldots ,\;N-1\right]$$

**Figure 3:**
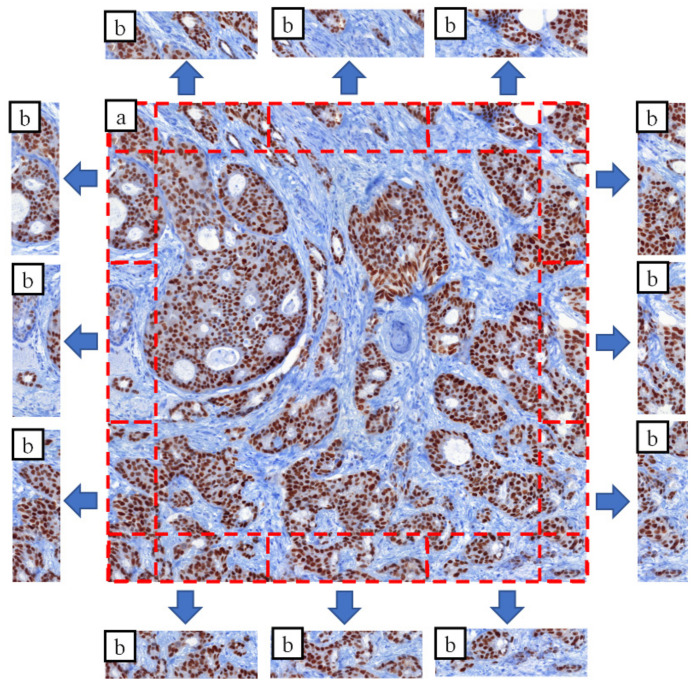
Patched based overlap division: (**a**) original tile; (**b**) patches extracted from overlap areas (4 × 3 patches); red lines: patches.

We extend the histogram matching for patch-based usage (*phm*)

(7) $$phm\left(p,\;i\right)=hm_{p}\left(i\right),\;\;\;\;\;\;i\in \left[0,\;\ldots ,\;N-1\right]$$

where *hm_p_* is calculated from the *p* patch using the *cdf_p_*_1_ and *cdf_p_*_2_ distribution functions. Figure 3 shows a field of view, its four overlap areas (top-left-bottom-right) and the patches created from them.

### 2.3. Executing Polynomial Regression

Along the overlapping areas usually there are intensities which is not contained by the overlapping pairs. However, transformation needs to be estimated since they can appear on the internal regions. This means we need to extrapolate and interpolate for these intensities.

Assuming the illumination inhomogeneities have the same characteristics for the whole range of intensities, we can estimate regression by the known intensities.

In general, polynomial regression is a form of regression analysis in which the relationship between the independent variable *x* and the dependent variable *y* is modelled as an nth degree polynomial in *x*. Polynomial regression fits a nonlinear relationship between the value of *x* and the corresponding conditional mean of *y*, denoted *E(y|x)*.

We can combine the patched based histogram matching with polynomial regression:

(8) $$x_{i}=i,\;\;\;\;\;\;i\in known\;intensities\;y_{i}=phm\left(i\right),\;\;\;\;\;\;i\in known\;intensities$$

The polynomial regression model

(9) $$y_{i}=\beta_{0}+\beta_{1}x_{i}+\beta_{2}x_{i}^{2}+\cdots +\beta_{m}x_{i}^{m}+\varepsilon_{i}$$

can be expressed in matrix form

(10) $$\left[\begin{array}{c} y_{1}\\ y_{2}\\ y_{3}\\ \vdots \\ y_{n} \end{array}\right]=\left[\begin{array}{ccccc} 1 & x_{1} & x_{1}^{2} & \cdots & x_{1}^{m}\\ 1 & x_{2} & x_{2}^{2} & \cdots & x_{2}^{m}\\ 1 & x_{3} & x_{3}^{2} & \cdots & x_{3}^{m}\\ \vdots & \vdots & \vdots & \ddots & \vdots \\ 1 & x_{n} & x_{n}^{2} & \cdots & x_{n}^{m} \end{array}\right]\;\left[\begin{array}{c} \beta_{1}\\ \beta_{2}\\ \beta_{3}\\ \vdots \\ \beta_{m} \end{array}\right]+\left[\begin{array}{c} \varepsilon_{1}\\ \varepsilon_{2}\\ \varepsilon_{3}\\ \vdots \\ \varepsilon_{n} \end{array}\right],$$

where $\vec{x}$ is the vector of known intensities, $\vec{y}$ is the vector of histogram matching, $\vec{\beta }$ is the vector of parameters, $\vec{ℇ}$ is the error vector. Using pure matrix notation:

(11) $$\vec{y}=X\;\vec{\beta }+\vec{ℇ}$$

The vector of polynomial regression coefficients ($\vec{\beta }$) can be estimated using ordinary least squares estimation (reference):

(12) $$\vec{\beta }={\left(X^{T}X\right)}^{-1}\;X^{T}\vec{y}$$

We can combine the patched based histogram matching with polynomial regression ($pr\left(p,i\right)$):

(13) $$pr\left(p,i\right)=\beta_{0}+\beta_{1}*i+\beta_{2}*i^{2}+\ldots +\beta_{n}*i^{n}$$

where $\vec{\beta }$ is calculated by the patch *p* and *n* is the length of polynomial function.

### 2.4. Applying the Compensation

We calculated regressions for each patch along the overlapping areas of tiles: one polynomial function for each patch. In order to apply the result of regression to the tiles first we need to define an interpolation function between the patches:

Let *lerp(a, b, t)* is the linear interpolation (*t* based) value between *a* and *b*:

(14) $$lerp\left(a,b,\;t\right)=a*\left(1-t\right)+b*t\;pri\left(x,\;i\right)=lerp\left(pr\left(p,i\right),\;pr\left(p+1,i\right),\;t\right)$$

where

(15) $$p=floor\left(x*N/W\right)\;t=mod\left(x/W*N,1.0\right)$$

where *W* is the width (or height) of tiles; *N* is the number of patches horizontally or vertically.

We are able to interpolate between the patches. Now, we can define a horizontal (*H*) and a vertical (*V*) compensation function:

(16) $$comp_{H}\left(x,\;i\right)=lerp\left(pri_{T}\left(x,i\right),\;pri_{B}\left(x,i\right),\;t\right)$$

where *T* and *B* are the top/bottom overlapping areas of a tile. Similarity we can define the vertical case:

(17) $$comp_{V}\left(y,\;i\right)=lerp\left(pri_{L}\left(y,i\right),\;pri_{R}\left(y,i\right),\;t\right)$$

where *L* and *R* are the left/right overlapping areas of a tile.

**Figure 4:**
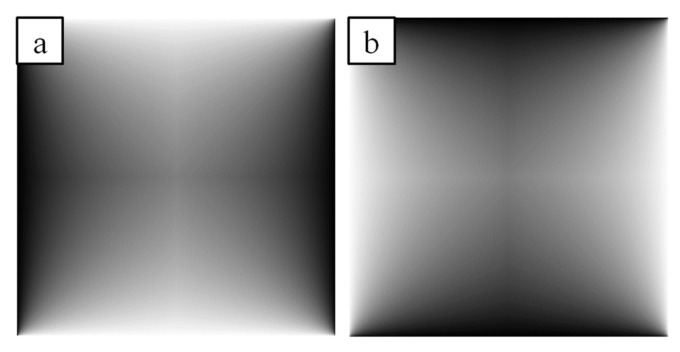
Interpolation weights: (**a**) horizontally; (**b**) vertically.

The following formula (*iw*) gives the linear interpolation weight (Figure 4.) for an *x,y* index between [0..1]:

(18) $$iw\left(x,y\right)=\frac{0.5-\left|0.5-\frac{x}{w}\right|}{1+\left|0.5-\frac{x}{w}\right|+\left|0.5-\frac{y}{h}\right|}$$

We can compose the vertical pairs of the above formulas similarly.

(19) $$comp\left(x,y,\;i\right)=\begin{array}{l} lerp(\\ comp_{H}\left(x,\;i\right),\\ comp_{V}\left(y,\;i\right),\\ iw\left(x,y\right)) \end{array}$$

To apply the interpolated adaptive histogram matching to the source image we use the following calculation:

(20) $$dst\left(x,y\right)=\frac{comp\left(\mathrm{src}\left(x,y\right),x,y\right)}{2}+\frac{src\left(x,y\right)}{2}$$

where *src*(*x,y*) is the intensity on the input image at *x,y* position and *dst*(*x,y*) is the compensated intensity in this position.

A brightfield scanning usually uses three channels (red, green, blue) while in case of fluorescent illumination the channel number can be up to ten or more. Applying the proposed compensation methods, we can compensate each channel independently [4,12,19].

### 2.5. Iterative Approach

After executing the compensation, the intensity differences along the borders of tiles are eliminated, however local brighter and darker tiles are still perceptible.

We can improve the algorithm using iterative approach to compensate the tiles considering their farther neighbors.

During the iteration we compensate using the above methods, but in each iteration, we use simulated annealing, when we spread the compensation values to the neighbors, with decreasing strength. Applying the proper iteration steps the tiles are compensated by not only their adjacent pairs, but by using farther tiles, reaching a more global optimum.

Iteration steps:

1. Calculate the histogram functions for each patch of overlap areas: *ph(i)*
2. Calculate the histogram matching for the patches: *phm(p,i)*
3. Estimate the polynomial regression for them: *pr(p,i)*
4. Condition:
  - Stop the loop after *N* iteration and continue with *Step 5*.
  - Calculate the average histogram matching values along the patches of each tile:
    (21) $$phm_{avg}\left(p,i\right)=\frac{\sum phm\left(p^{\prime },i\right)}{N}$$
    where *p’* are the patches of the tile and *N* is the number of patches in a tile.
  - Spread the average histogram matching with decreasing strength (*w*):
    (22) $$phm\left(p,i\right)=lerp\left(phm\left(p,i\right),\;phm_{avg}\left(p,i\right),\;w\right)$$
  - Continue with the *Step 3*.
5. Compensate the tiles using the compensation function: *comp(x,y,i)*

**Figure 5:**
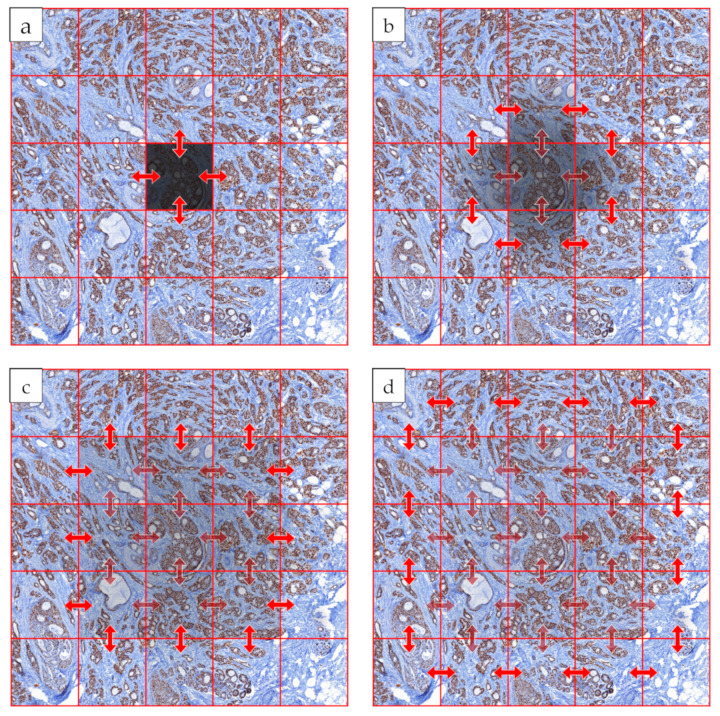
Illustration of iterative steps: (**a**) first iteration; (**b**) second iteration; (**c**) third iteration; (**d**) fourth iteration; light red arrows: compensations in the step.

On Figure 5, it is shown how a darker field of view is eliminated during the iteration steps. The arrows only illustrate compensations started from the middle field of view during the iterations, in fact, in each iteration, all fields of view are compensated by their neighbors. After the first iteration, the neighbors of the middle FOV became darker, but after a few iterations, the darkness can be eliminated: the fault of the middle FOV disappears without spoiling its neighborhood.

### 2.6. Multi-Focal Compensation

When we compensate the layers of a multi-focal scanning independently one-by-one, we get homogeneous 2-dimensional layers. However, when we compare these layers with each other, we see that the intensities along the focal layers (*Z* layers) are different. It is possible to enhance the method with inter-layer compensation.

Take the case when we scan the slides in *Z-X-Y* order: the scanner moves to a specific *X-Y* position, scans each focal layers, and then moves to the next position. In this case the overlapping areas are the same which we can take advantage for compensating between the layers.

**Figure 6:**
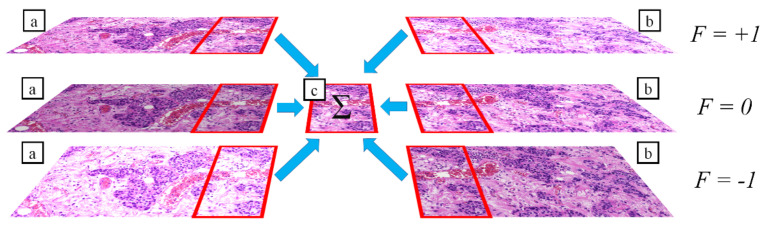
Illustration of multi-focal compensation: (**a**) left neighbors of a FOV; (**b**) right neighbors of a FOV; (**c**) compensated result of the six adjacent images; red rectangles: overlapping areas.

In this case an overlapping area not only has left and right (or above/below) pairs, but it has pairs also in *Z* order (Figure 6.). The idea is to use the Z neighbors in the “spreading” step of the iterative approach (4. b. step):

(23) $$ph{m_{avg}}^{\prime }\left(p,i\right)=\frac{\sum phm\left(p^{\prime \prime },i\right)}{N^{\prime }}$$

where *p*′′ are the patches of the tile in horizontal/vertical and Z direction and *N’* is the number of patches these patches.

## 3. Validation

The validation process was done by using 10 slides of 10 clinical cases. The slide set was selected based on image quality. Only slides with tile effect issues were selected by using manual investigation. 1200 FOVs were used from the selected slide set with an average of 2-megapixel-FOV-size. The methods were executed native resolution.

Three different aspects were investigated in the validation protocol:

1. Average intensity error *(error)*
2. Heterogeneity of tile intensities *(heterogeneity)*
3. Intensity error between focal layers *(zerror)*

### 3.1. Average Intensity Error

The average intensity error (*error*) was measured in each tile, that was calculated based on the average intensity differences in the overlapping area of the tiles (*error_a,b_*):

*error_a,b_* is the average intensity error of a tile-pair:

(24) $$error_{a,b}=\frac{\sum_{x,y}^{}\left|o_{a,b}\left(x,y\right)-o_{b,a}\left(x,y\right)\right|}{n}$$

where *a* and *b* are two FOVs, *x* and *y* are the pixel coordinates on the overlap areas, *n* is the number of pixels and *o_a,b_* is the pixels of overlap area of the FOV *a* with the FOV *b*.

(25) $$error=\frac{\sum_{c}^{}\sum_{a,b}^{}error_{a,b}}{n_{c}*\sum_{c}^{}n_{c,a,b}}$$

where *c* is the case number, *a,b* is a tile-pair of the case *c*, *n_c_* is the number of cases and *n_c,a,b_* is the number of tile-pairs of case *c*.

### 3.2. Heterogeneity of Compensation

One aim of the optimizations is to compensate the tiles in a way that make them look homogenous. We calculated the differences under the overlapping areas (*error*), but we need to measure the heterogeneity between the tile intensities.

Let *avg_a_* is the average intensity of the pixels of tile *a*:

(26) $$avg_{a}=\frac{\sum_{x,y}^{}src_{a}\left(x,y\right)}{n}$$

where *a* is a tile, *x* and *y* are the pixel indices on the tile *a* and *n* is the number of pixels of tile.

Let *diff_a,b_* is the absolute difference of average intensities of two neighboring tiles:

(27) $$diff_{a,b}=\left|avg_{a}-avg_{b}\right|$$

where *a* and *b* are adjacent tiles (horizontally or vertically).

Let *diff^o^_a,b_* is the original (uncompensated) difference of *a* and *b* tiles, and let *diff^c^_a,b_* is the compensated difference.

Let *heterogeneity* is average difference ratio between the compensated (*diff^c^_a,b_*) and uncompensated (*diff^o^_a,b_*) tiles:

(28) $$heterogeneity=\frac{\sum_{c}^{}\sum_{a,b}^{}\left(\frac{diff^{c}{}_{a,b}}{diff^{o}{}_{a,b}}\right)}{n_{c}*n_{c,a,b}}$$

where *c* is the case index, *a,b* is a tile-pair of the case *c*, *n_c_* is the number of cases and *n_c,a,b_* is the number of tile-pairs of case *c*. This formula gives relative heterogeneity for the differences: before the compensation this value is 1.0 and the compensation needs to decrease this value. A lower value means that the differences after the compensation are lower.

### 3.3. Intensity Error between Focal Layers

We can extend the error function for analyze the differences between focal layers using their overlap areas:

*zerror_a,b_* is the average intensity error of a tile-pair which are located one above the other:

(29) $$zerror_{i,j,}=\frac{\sum_{x,y}^{}\left|o_{i,j}\left(x,y\right)-o_{i,j}\left(x,y\right)\right|}{n}$$

where *i* and *j* are two FOVs in a neighboring focal layers, *x* and *y* are the pixel coordinates on the overlap areas, *n* is the number of pixels and *o_i,j_* is the pixels of overlap area of the FOV *i* with the FOV *j*.

(30) $$zerror=\frac{\sum_{c}^{}\sum_{i,j}^{}zerror_{i,j}}{n_{c}*\sum_{c}^{}n_{c,i,j}}$$

where *c* is the case number, *i,j* is a tile-pair of the case *c*, *n_c_* is the number of cases and *n_c,i,j_* is the number of tile-pairs of case *c*.

## 4. Results

**Figure 7:**
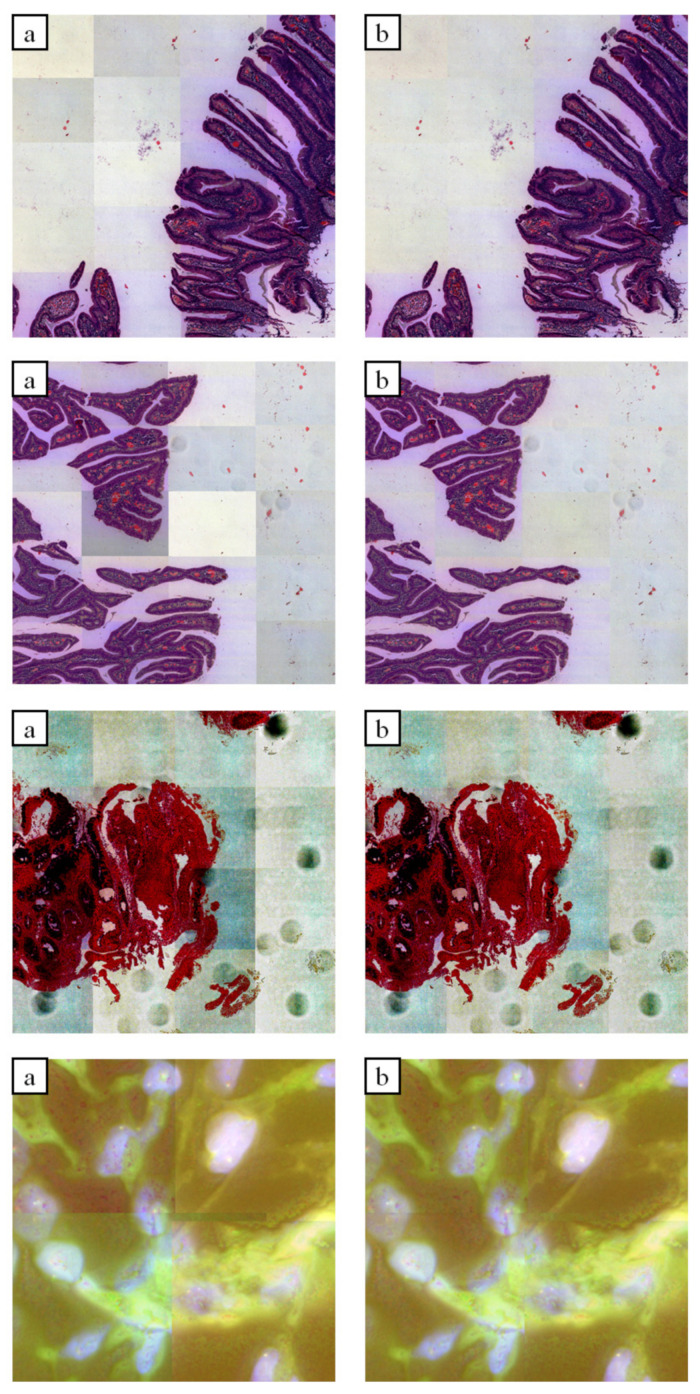
Results of iterative approach: (**a**) input images; (**b**) compensated images.

**Figure 8:**
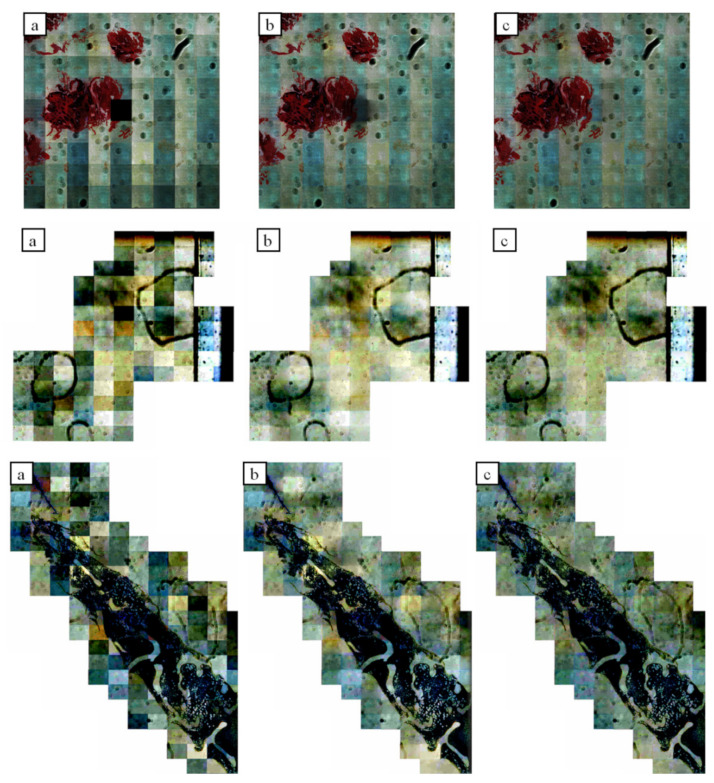
Results of iterative approach: (**a**) input image; (**b**) compensated image without iterations; (**c**) iterative compensation.

The Figure 7 and Figure 8, illustrations show the differences between the original and iteratively compensated images along the edges of tiles. To make the differences easier to see on these illustrations, their contrast has been increased.

**Figure 9:**
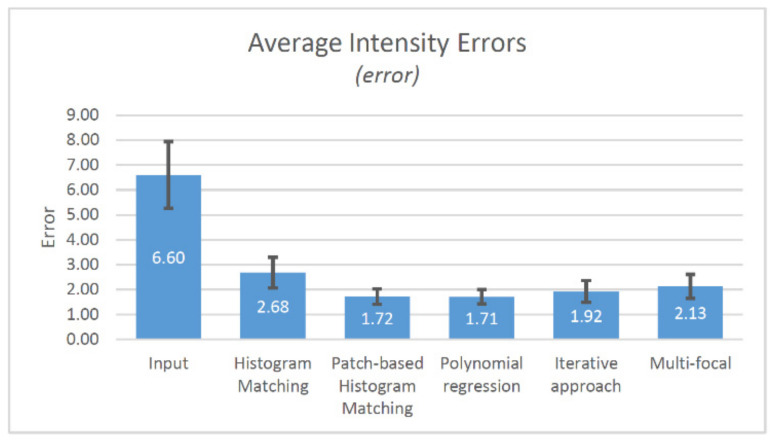
The results of the intensity error measurement (average and std. dev.).

Table 1 shows the validation results of five algorithms analyzing them with three metrics. The average value and standard deviation are displayed for results. Regarding the two-dimensional average compensation error (Figure 9.), applying histogram matching-based compensation, more than two-times less error (2.68) has been found compared to the input image (6.60). The patch based (1.72) and regression based (1.71, second-order polynomial) methods result quite the same errors. The iterative method (1.92) and multi-focal approach (2.13) gave a bit more errors compared with the regression-based method. We use second-order regression, since the samples are almost linear (first order), but they contain exponential-like aberration. We founded the third-order regression unnecessary. We choose three patches since most of the unevenness homogeneous inside the field of views, only a little difference can be observed in the corners of tiles which are not larger than one third of the width of the field of views.

**Figure 10:**
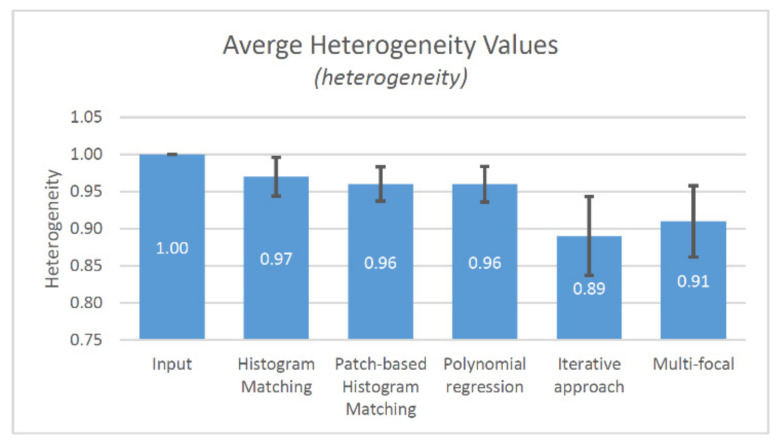
The results of the heterogeneity measurement (average and std. dev.).

Analyzing the heterogeneity (Figure 10), we can see that except the iterative approach (0.89) the other methods give almost the same results (0.9×). It is not surprising; the iterative approach was made to eliminate the inter-tile heterogeneity. We use five iteration steps for the validation set. The number of needed iterations is greatly determined by the characteristics of sample, since using lower iteration number, the compensation is not able to compensate a local unevenness, but applying larger iteration count the possibility of compensating the not related tissue areas to each other are increasing. For the validation set five iteration is enough.

**Figure 11:**
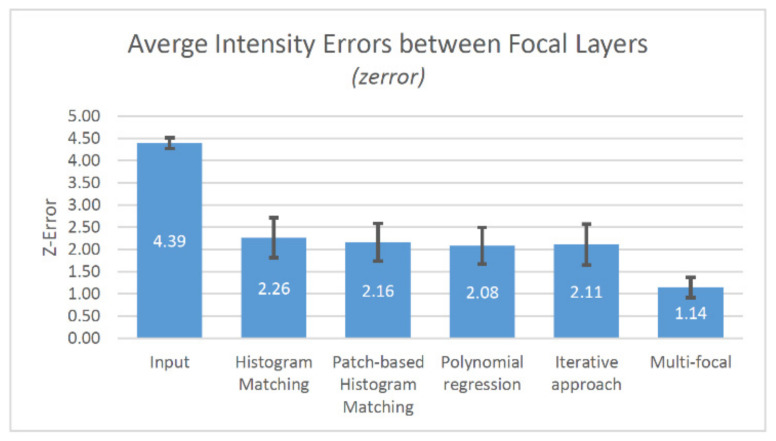
The results of the focal layer error measurement (average and std. dev.).

Examining the errors between the focal layers (Figure 11), the input images had value of 4.39. It is more than twice as applying histogram (2.26), patch based (2.16), regression based (2.08) or iterative (2.11) approaches. However, the multi-focal optimization gave the best results (1.14) for z-error.

## 5. Discussion

The purpose of this study was to investigate how different histogram matching-based compensation methods perform in the task of eliminating the illumination differences in a whole slide imaging system. The present results suggests that the most accurate compensation can be obtained by the patch-based polynomial regression method extended with the iterative and multi-focal approaches, however, all five methods have its own benefits.

The methods are designed to reduce the perceptible intensity differences along the tile boundary. In general, using more sophisticated algorithms to do the compensation, the probability of causing undesirable artefacts at the middle of the tiles are increasing. The simplest histogram matching-based method calculates the compensation factor about the four overlapping areas of field of views, so doing a false compensation is minimal. The other end is the iterative patch-based approach, where the more parts we divide the overlapping area, the more we can reduce the differences at the tile boundaries, but the chances increase that we need to compensate intensities at the tile center which are not represented in the overlapping areas. In such cases, we need to extrapolate excessively.

All five proposed methods have their own characteristics, from which we need to choose one that is flexible enough to correct the uneven intensities. For instance, when the inhomogeneities in our WSI system are caused by the stochasticity of the illumination, whose unevenness is linear and homogeneous throughout the field of views, the histogram matching method could be appropriate. However, if the strength of the inhomogeneities are different in parts of the field of view, we need to use a more adaptive approach. For some scanner configurations, it can be observed that the illuminating stochasticity is not completely random, only some FOVs are darker than others. In this case, using the iterative extension, we can eliminate these darker areas, since the local differences became visible to the algorithm considering wider neighborhood. Multi-focal extension can compensate the intensity differences between layers in multi-focus scanning, however, it should be noted that not all focus layers contain exactly the same tissue structure, they can be visually different, and its extent is depending on the sample thickness and distance between layers. If the layer distance is greater (e.g., greater than a few microns), there is an increased chance of compensating by independent tissue structures.

The present study validates the field of views before and after the execution of compensation mostly by synthetic methods. There are two main reasons why we want to produce images with as little unevenness as possible: to make the doctor’s job easier and to improve the reliability of automatic and algorithmic evaluation. In both cases, the visible and detectable intensity differences decrease the accuracy. No matter how adaptive or intensity-independent is the processing, be it a traditional algorithm or even a more sophisticated convolutional neural network, if it is unaware that there can be intensity errors at a certain point of the slide, it will detect incorrectly. The effect of compensation on human and machine measurements and processing could be examined in a future study.

Analyzing the results of the validation set, several ideas have been put forward to improve the proposed methods. One such optimization is when there is just a little information in an overlapping area, such as when it only contains background intensities. Then, there is a greater chance that the real tissue structure in the middle of the field of view can be compensated using a large-scale extrapolation. However, it can be detected, for example, if the intensities in the middle of FOV are considered by how much they deviate from the overlapping area. If the discrepancy is large, we need to reduce the strength of local compensation and take better account of the wider neighborhood in the iterative approach.

Another improvement possibility is the dynamic determination of the number of iterations, which is currently defined and fixed at the beginning of the model execution. In case of a larger number of iterations, there is a chance to do the compensation by a more distant area that does not belong to the same tissue structure as the reference field-of-view, causing incorrect compensation. Recognition of different tissue structures is a well-studied research field in the literature, with many approaches and methods already available from traditional pattern recognition algorithms [31] to sophisticated, deep learning based convolutional neural network methods [32,33,34].

In addition to digital microscopy, the proposed methods can be adapted to other disciplines, in which images must be compensated to each other, improving the quality and consistency of images or samples. The compensated images may be side by side, such as in case of panoramic photography or satellite pictures, may follow one another in time, such as in a motion picture or in astronomical images, or even spatially behind each other, such as in optical coherence tomography or magnetic resonance imaging. The field of application can be very diverse, and the proposed methods are flexible enough to be transferred with little modifications.

## 6. Conclusions

The use of tile compensation methods is critical in whole slide imaging (WSI). In this study we collect a set of digital slides with tile effect issues and after the implementation of five different compensation methods we have made a validation study to determine which models provides the best results.

According to the characteristics of the collected validation set, the polynomial regression method gave the best results regarding the average compensation error validation, however considering the tile homogeneity, the iterative method can provide better results. In the case of multi-layer slides, the multi-focal optimization can enhance the quality of compensation.

Finally, we can conclude that each approach can be useful for compensation of FOV in whole slide imaging systems, but in our validation study, we find the iterative approach most promising using the multi-focal extension for multi-layer slides.

## Figures and Tables

**Table 1:** The compensation results of different methods.

Method	Intensity Error *(error)*		Heterogeneity *(heterogeneity)*		Focal Error *(zerror)*	
	Average	Std. Dev.	Average	Std. Dev.	Average	Std. Dev.
Input image	6.60	1.34	1.00	0.000	4.39	0.12
Histogram Matching	2.68	0.62	0.97	0.026	2.26	0.45
Patch-based Histogram Matching	1.72	0.31	0.96	0.023	2.16	0.42
Polynomial Regression	1.71	0.29	0.96	0.024	2.08	0.41
Iterative Approach	1.92	0.44	0.89	0.053	2.11	0.46
Multi-focal Approach	2.13	0.48	0.91	0.048	1.14	0.23

## Data Availability

Data used in this study can be made available upon reasonable request.
